# ScRNA-seq revealed the kinetic of nasopharyngeal immune responses in asymptomatic COVID-19 carriers

**DOI:** 10.1038/s41421-021-00294-x

**Published:** 2021-07-27

**Authors:** Furong Qi, Gang Xu, Xuejiao Liao, Fuxiang Wang, Jing Yuan, Haiyan Wang, Xin Wang, Lei Liu, Shuye Zhang, Zheng Zhang

**Affiliations:** 1 Institute for Hepatology, National Clinical Research Center for Infectious Disease, Shenzhen Third People’s Hospital; The Second Affiliated Hospital, School of Medicine, Southern University of Science and Technology, Shenzhen, Guangdong China; 2 Department for Infectious Diseases, Shenzhen Third People’s Hospital, Shenzhen, Guangdong China; 3 Shenzhen Research Center for Communicable Disease Diagnosis and Treatment of Chinese Academy of Medical Science, Shenzhen, Guangdong China; 4 Shanghai Public Health Clinical Center, Fudan University, Shanghai, China; 5 Shenzhen Bay Laboratory, Shenzhen, Guangdong China; 6 Guangdong Key Laboratory for Anti-infection Drug Quality Evaluation, Shenzhen, Guangdong China

**Keywords:** Innate immunity, Bioinformatics

Dear Editor,

Asymptomatic (or silent) severe acute respiratory syndrome-coronavirus-2 (SARS-CoV-2) infections are unique and curtail the control of viral transmission; however, it is unclear how the immune system of asymptomatic carriers reacts particularly when it comes to clearing the virus and sparing the host from any symptoms. Here, we revealed the kinetics of nasopharyngeal immune responses in asymptomatic carriers using single-cell transcriptomic RNA sequencing (scRNA-seq). A unique immune homeostatic environment was found in the nasopharynx locally in asymptomatic infections, including strengthening of epithelial compartments, sequential transition of monocyte/macrophage functions, vigorous CD8^+^ T-cell clonal expansion. This supports that a rapid and coordinated immune defense at the respiratory barrier site is the key to the successful control of SARS-CoV-2 infections.

The coronavirus disease 2019 (COVID-19) pandemic is one of the most serious public health crises in human history. The clinical manifestations of COVID-19 are diverse, ranging from asymptomatic infections, mild symptoms, to viral pneumonia and life-threatening acute respiratory distress syndrome^1^. Asymptomatic infections, such as patients who are infected with SARS-CoV-2 but show no symptoms, are very common but hard to identify, making efforts to stop transmission almost impossible^2,3^. Although previous studies have revealed some aspects of immune responses in symptomatic COVID-19 patients^4,5^, little is known about the immune responses especially in the upper respiratory mucosal barriers where the SARS-CoV-2 infection starts.

In this study, scRNA-seq was used to profile the nasopharyngeal swabs of five asymptomatic COVID-19 carriers, at both the early-stage (AA, within 3 days after PCR positivity confirmation) and late-stage (AP, within 6 days after PCR turned negative) of the infection. The publicly available scRNA-seq data of nasopharyngeal swabs from 8 mild (M) and 11 severe patients (S) and 6 healthy controls (H) were also evaluated^6^ (Fig. 1a). In total, 97,251 cells (Supplementary Fig. S1a and Table S1) passed the stringent quality control and were categorized into 9 cell lineages, including epithelial cells (club, ciliated, and squamous), T cells (CD8-GZMB and CD8-RPS27), B cells, neutrophils, and macrophages (Mφ-FCN1 and Mφ-APOE) based on their specific markers (Fig. 1b).

**Fig. 1:**
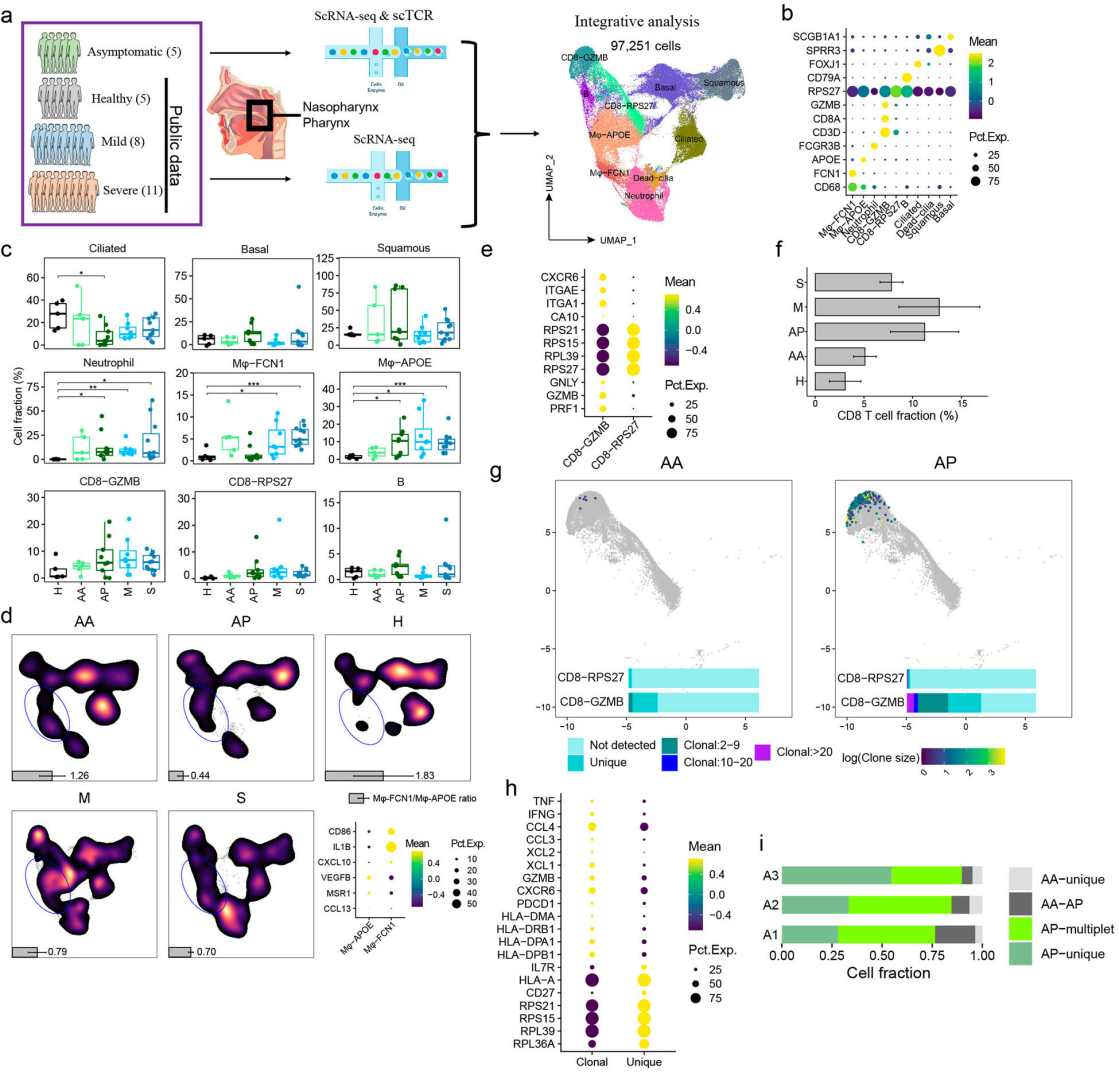
Characterizing nasopharyngeal immune landscapes in asymptomatic and symptomatic SARS-CoV-2 infections. **a** Illustration of the studied subjects and experimental scheme. **b** The canonical markers defining the nine major cell types. **c** Percentages of the nine cell types in nasopharyngeal swabs from healthy controls (H), early (AA) and late (AP) samples of asymptomatic carriers, the mildly (M) and severely (S) ill COVID-19 patients. (Two-sided *t* test. ^*^P < 0.05; ^**^P < 0.01; ^***^P < 0.001). **d** UMAP density plots showing the distribution of macrophages subtypes identified by inflammatory or anti-inflammatory markers (bottom right panel). Gray bars indicate the ratio of Mφ-FCN1/Mφ-APOE. **e** Expression of selected marker genes by CD8-GZMB and CD8-RPS27. **f** The proportion of total CD8^+^ T cells from each studied group. **g** The TCR clonal expansion status of the nasopharyngeal CD8^+^ T cells. **h** Selected differentially expressed genes by expanded vs non-expanded CD8^+^ T cells. **i** Following the “AA-to-AP” TCR dynamics in three asymptomatic individuals (AA–AP indicates TCR-sharing clonotypes between AA and AP; singlet and multiplet indicate non-sharing non-expanded or expanded TCR clonotypes, respectively).

Of the five enrolled asymptomatic carriers, none self-reported any symptoms, which was also verified by the examining results of the common clinical manifestations. All clinical endpoints throughout the study displayed near-normal values, including C-Reactive Protein (CRP), interleukin 6 (IL-6), lactate dehydrogenase (LDH), D-dimer, and blood cell counts (Supplementary Table S2). There were low levels of viral RNA (high Ct values) in the early-stage of asymptomatic infection, as identified through nasopharyngeal swabs, whereas viral RNA turned negative in the late-stage samples. Examining the scRNA-seq data, we also found that viral RNA was undetectable in asymptomatic cases (Supplementary Fig. S1b), possibly due to the low viral load in these carriers. By contrast, the viral RNA was present in swabs from mild and severe COVID-19 patients (Supplementary Fig. S1c).

Epithelium is the first barrier against invading pathogens^7^. It is unclear, however, what roles epithelial cells play in the defense against SARS-CoV-2 infections. The key host genes *ACE2*^8^, *TMRPSS2*^9^, *NRP1*^10^, and *NRP2*, which mediate SARS-CoV-2 infection, are expressed by epithelial cells and upregulated in those with symptomatic vs asymptomatic infections (Supplementary Fig. S1c, d). Three types of epithelial cells were identified and analyzed in this study. They have an intriguing division of labor. For example, the genes related to immune responses including neutrophil degranulation and NF-κB signaling (*ECM1, IL1RN, NDFIP2, NFKBIA*, etc.), were specifically enriched in squamous cells (Supplementary Fig. S1e, f). The ciliated cells show signatures of cilium-related functions, and Basal cells, as potential progenitors, show metabolism-related signatures (Supplementary Fig. S1e, f). Both ciliated and basal cells were decreased in COVID-19 patients (Fig. 1c). Notably, the barrier functions of ciliated (cilium assembly) and squamous cells (epiderm differentiation and cytokine production) were strengthened in asymptomatic infections, while there was an increase of type I IFN-activated signature in symptomatic infections (Supplementary Fig. S1g).

Besides the epithelium remodeling, the nasopharyngeal immune cellular compartments in asymptomatic carriers differed significantly from those in healthy controls and symptomatic cases. For example, there was increased infiltration of immune cells, including neutrophils, macrophages, and T lymphocytes compared to healthy controls (Fig. 1c). Pronounced neutrophil functionality indicated by higher levels of neutrophil degranulation genes, and IL-1, IL-12, and IFN-γ responses were observed in severe COVID-19 patients. In contrast, neutrophil activity was more confined in asymptomatic carriers and mild COVID-19 patients (Supplementary Fig. S2a, b). It is unclear whether the increased infiltration of neutrophils plays any anti-viral roles.

Nasopharyngeal macrophages were also analyzed and consisted of two subtypes: Mφ-FCN1 expresses *CXCL10*, *IL1B*, and *CD86* as the pro-inflammatory M1-type, and Mφ-APOE expresses *CCL13*, *MSR1*, and *VEGFB* as the anti-inflammatory M2-type (Fig. 1d). The ratios of M1 and M2 macrophages changed sequentially in asymptomatic infections. The early-stage was dominated by the M1 (1.26-fold of M1/M2 ratio), while the later stage was dominated by the M2 macrophages (0.44-fold of M1/M2 ratio) (Fig. 1d). By analyzing macrophage transcriptomes, it was found that the genes related to the cellular response to lipopolysaccharide and multiple chemokines were highly expressed in AA, implying that innate immunity was primarily activated at the AA stage of viral infection. Whereas the genes involved in macrophage activation and T-cell receptor signaling pathway were elevated in AP (Supplementary Fig. S3a, b). This signifies a transition from pro-inflammatory to tissue-repairing functions in those recovering from an acute respiratory infection. Only low levels of cytokines (IL-6, IL-10, IL-12, and IL-17A) were detected in plasma from asymptomatic patients (Supplementary Fig. S3c and Table S3), in consistence with lower levels of nasopharyngeal inflammation. However, severe patients expressed elevated transcripts related to response to the virus, neutrophil degranulation, response to IL-1 and Fc receptor signaling pathway (Supplementary Fig. S3d). The nasopharyngeal macrophages in those severe COVID-19 patients produced higher levels of chemokines (*CCL2, CCL3, CCL4, CXCL1, CXCL2, CXCL3*), indicating their roles in maintaining the cytokine storm (Supplementary Fig. S3e).

We also found two subsets of nasopharyngeal T cells, the CD8-GZMB and CD8-RPS27. CD8-GZMB subset expressed tissue-residency markers *CXCR6, ITGA1, ITGAE,* and effector genes *GZMB, GNLY, PRF1*, therefore identifying them as the tissue-resident T cells with effector functions. And the CD8-RPS27 subset highly expressed ribosomal genes but lacked typical effector molecules (Supplementary Fig. S4a and Fig. 1e). We found increased nasopharyngeal CD8^+^ T cells infiltrates from early to late-stage of asymptomatic infections (Fig. 1f). Sc-TCR analysis revealed much higher clonal expansion of CD8-GZMB cells in AP versus AA (Fig. 1g), suggesting that viral infection triggered an adaptive immune response at the AP stage. Clonally expanded CD8^+^ T cells expressed higher levels of *IFNG, TNF, HLA-DR*, and *CXCR6* (Fig. 1h) than their non-clonal counterparts, supporting that expanded nasopharyngeal CD8-GZMB cell clones reacted against SARS-CoV-2 infection. Examining the sequential samples individually, newly clone-expanded CD8-GZMB T-cell clones (with unique, non-sharing TCR clonality) were continuously present at the mucosal barriers during asymptomatic infections (Fig. 1i), probably as the key reinforcement to clear the virus and strengthen the barrier defense.

Finally, to further understand the nasopharyngeal immune microenvironment in asymptomatic COVID-19, the cell–cell interaction network was examined through analyzing receptor-ligand pairs among squamous, neutrophils, macrophages, and CD8-GZMB cells. Healthy controls were omitted due to the scarcity of immune cells. Macrophage cross-talking with other cells is the most prominent, and severe patients distinguished other cases by the enhanced neutrophil crosstalk (Supplementary Fig. S4b). Several signaling partners, including multiple chemokine receptors (*CXCL1/3-CXCR1/2, CCL7-CCR1, CCL3-CCR1*), are specifically upregulated between macrophages and neutrophils in patients experiencing severe COVID-19 (Supplementary Fig. S4c). In contrast, the enhanced interactions between squamous epithelial cells and macrophages in asymptomatic individuals were identified, including *MIF-CD74, IL1RN-IL1R, AREG-ICAM1*, and *TNF/CCL4L2-VSIR* (Supplementary Fig. S4d). These cells have been previously reported to participate in immune inhibition and repair, suggesting a unique immune homeostatic environment at mucosal barriers during asymptomatic SARS-CoV-2 infection.

In summary, this study provides the first comprehensive transcriptional and cellular landscape of the nasopharyngeal mucosa in asymptomatic COVID-19 carriers. These findings highlight an optimal immune response effectively clearing the virus without causing diseases, characterized by enhanced epithelium barrier function, mild inflammation, and robust CD8^+^ T-cell response locally.

## Supplementary informationś

Supplementary Figures and Methods

Supplementary Table S1

Supplementary Table S2

Supplementary Table S3
