# Supplementary material for: ScRNA-seq revealed the kinetic of nasopharyngeal immune responses in asymptomatic COVID-19 carriers
Source: Cell Discov. 2021 Jul 27;7:56. doi: 10.1038/s41421-021-00294-x (PMC8314853; doi:10.1038/s41421-021-00294-x)
Supplement: Supplementary file 1 — Supplementary Figures and Methods [file 41421_2021_294_MOESM1_ESM.pdf]

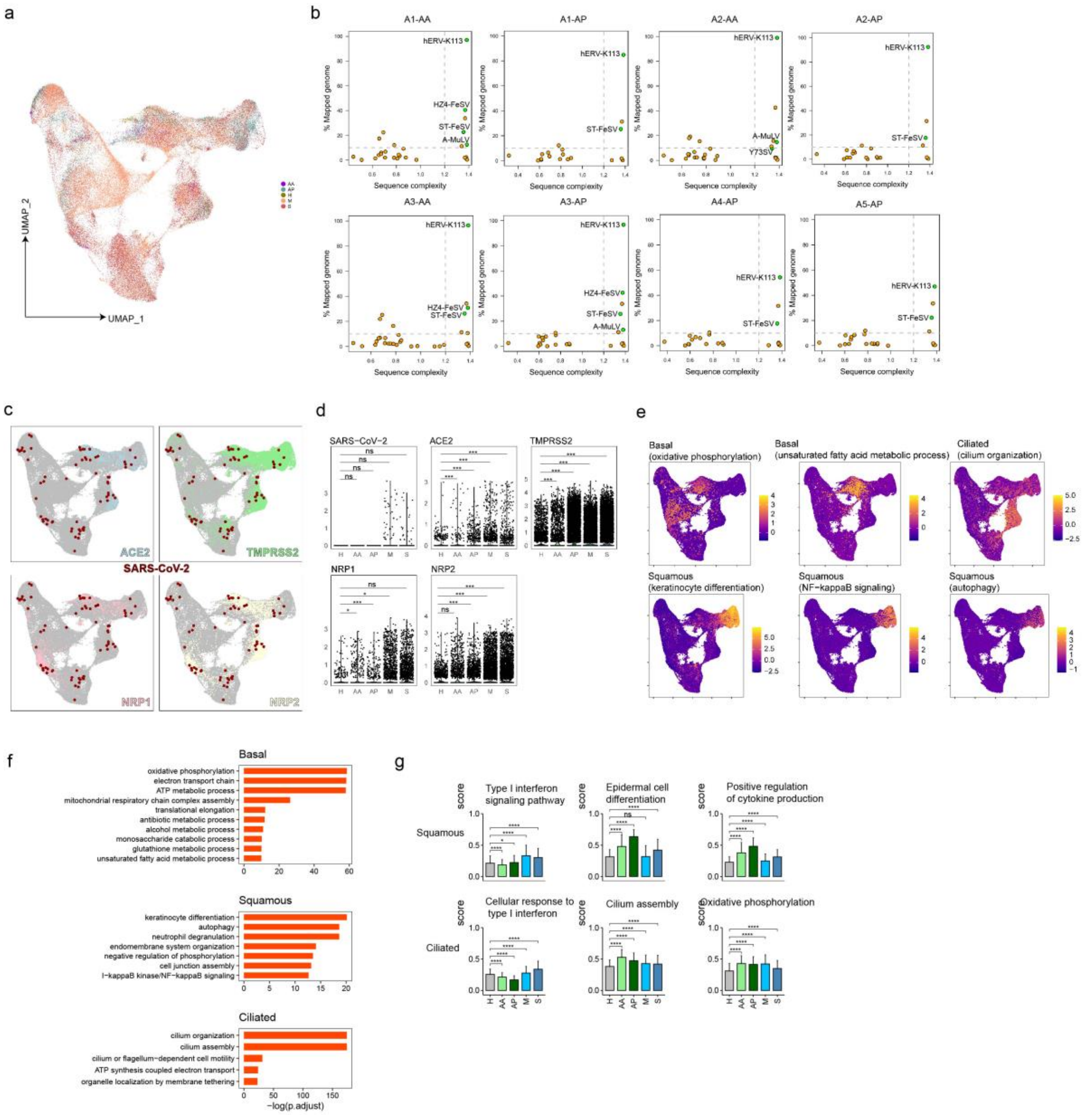

Supplementary Fig. S1 Examining SARS-CoV-2 infections and nasopharyngeal epithelial cell types. a Cell sources from different groups of subjects (H, AA, AP, M, S). **b** Viral-Track analysis performed on asymptomatic samples. **c** UMAP plots of SARS-CoV-2 reads and viral receptors (*ACE2, TMPRSS2, NRP1 and NRP2*). **d** Expression levels of SARS-CoV-2 reads and viral receptors (*ACE2, TMPRSS2, NRP1 and NRP2*) in all cells from each studied group. (Two-sided t test. $^{*}P < 0.05$; $^{**}P < 0.01$; $^{***}P < 0.001$; ns, not significant). **e** UMAP plots of functional features associated with basal, ciliated and squamous epithelial cells. **f** Selected Gene Ontology (GO) biological process (BP) terms of upregulated differentially-expressed genes by basal, ciliated and squamous epithelial cells. **g** Selected signatures of squamous and ciliated epithelial cells.

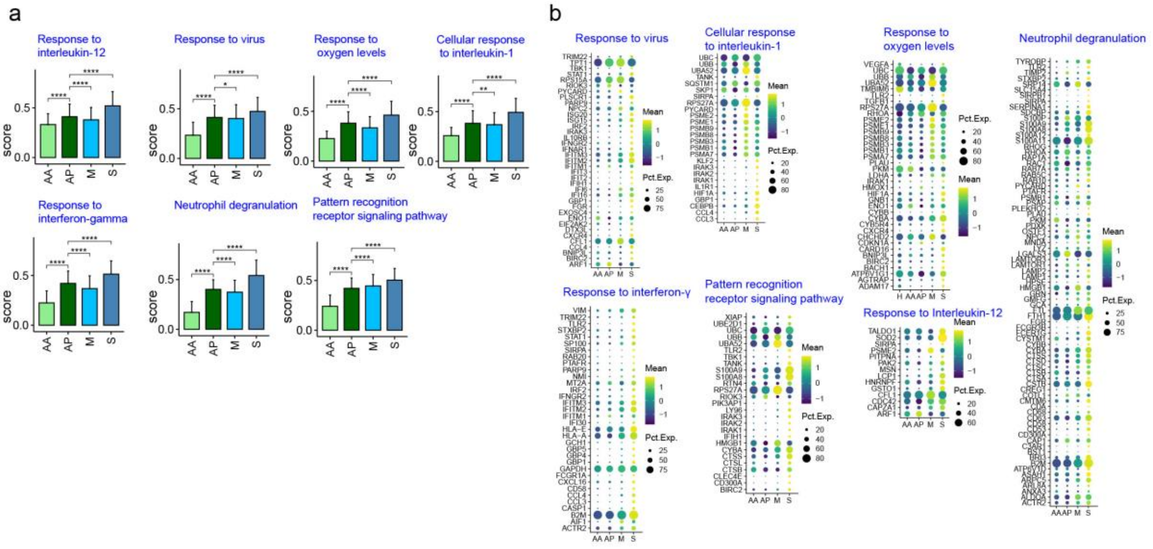

Supplementary Fig. S2 Transcriptome analysis of neutrophils in asymptomatic and symptomatic infections. a Selected functional neutrophil signatures from SARS-CoV-2 infection groups (Two-sided t test. $^{*}P < 0.05$; $^{**}P < 0.01$; $^{****}P <$ 1e-04). **b** The dot plot of differentially-expressed genes (DEGs) constituting the signature scores in "a".

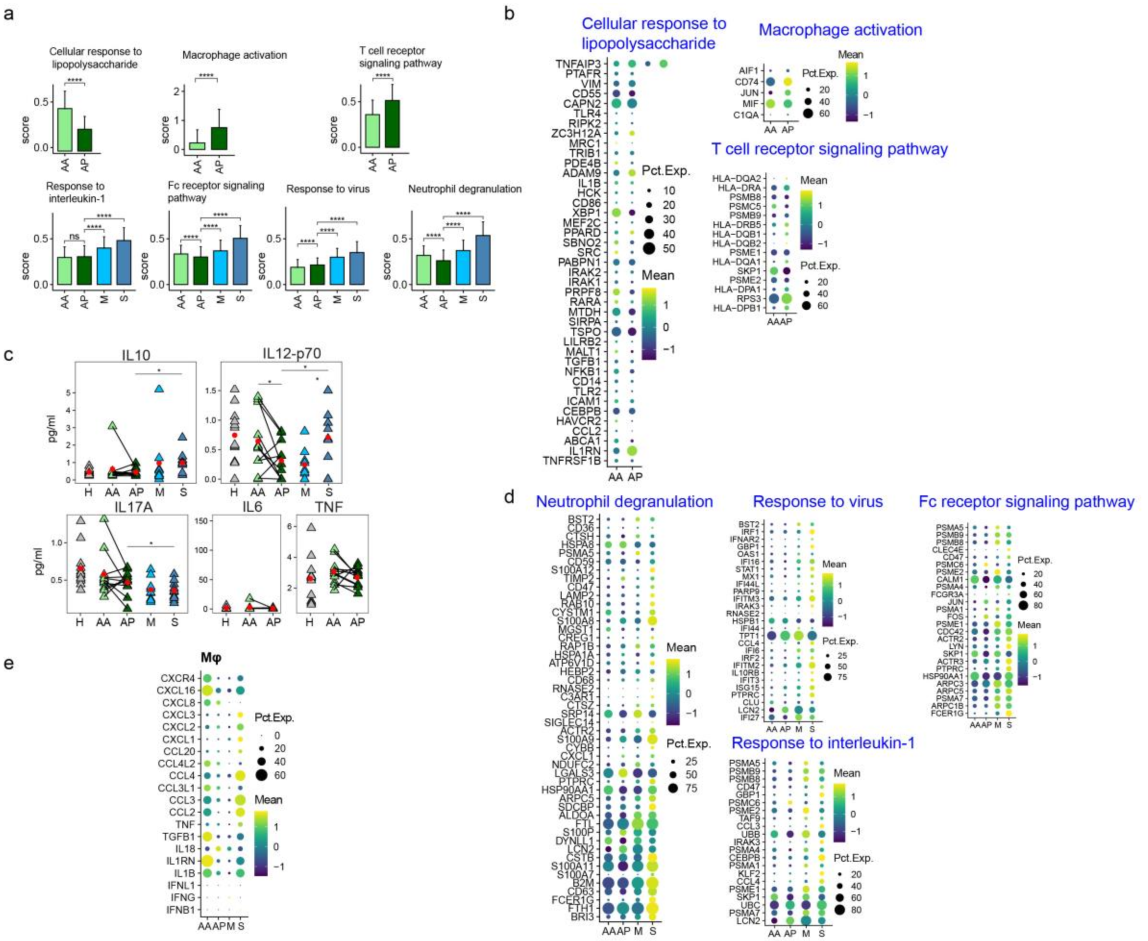

Supplementary Fig. S3 Transcriptome analysis of macrophages in asymptomatic and symptomatic infections. a Selected signatures of macrophages comparing AA and AP and across asymptomatic and symptomatic infections (Two-sided t test. Ns, not significant; $^{*}P < 0.05$; $^{****}P < 1e\text{-}04$). **b** The dot plot of DEGs related to the upper panel of "a". **c** The plasma levels of selected cytokines and chemokines measured by Meso Scale Discovery (MSD) Electrochemiluminescence assays (Two-sided t test. $^{*}P < 0.05$). **d** The dot plots of DEGs related to the lower panel of "a". **e** The dot plots of cytokines/chemokines genes in asymptomatic and symptomatic infection groups.

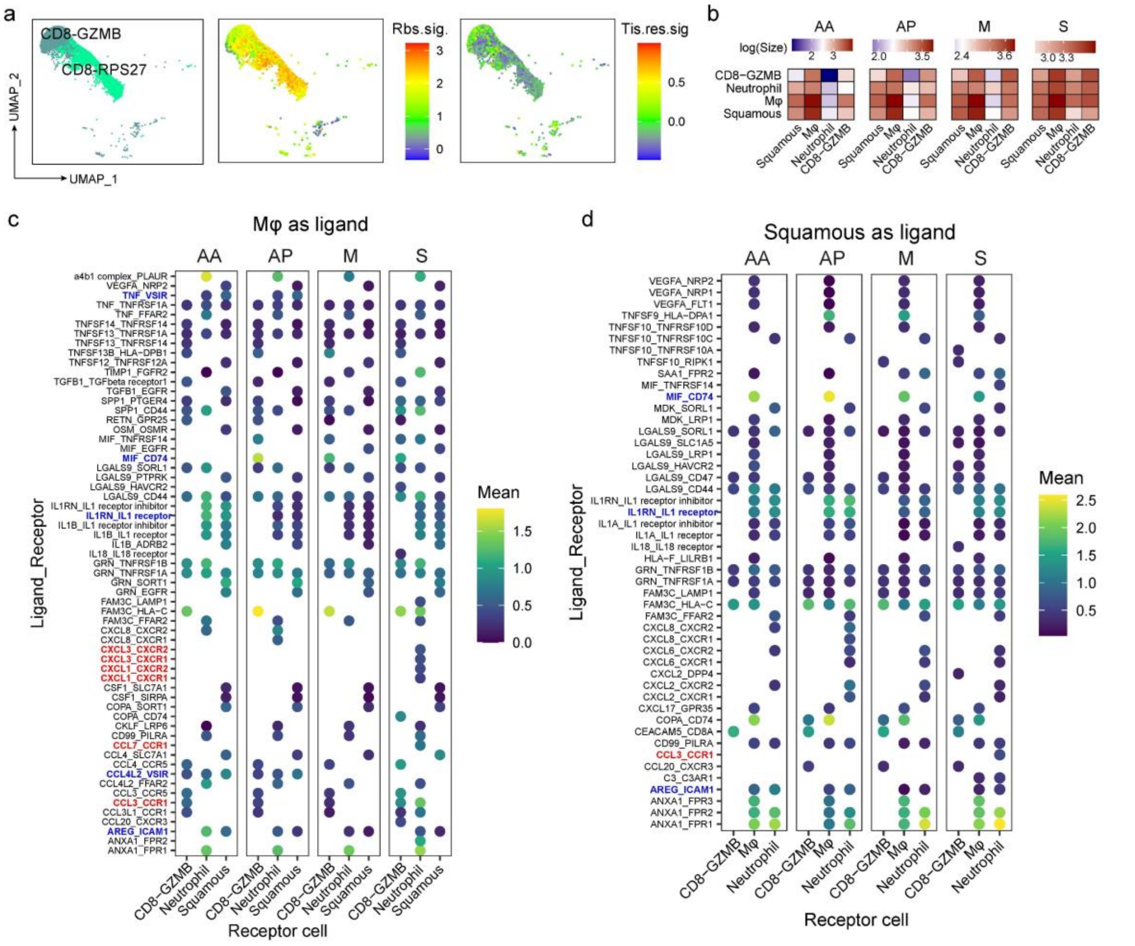

Supplementary Fig. S4 Nasopharyngeal cell-cell interactions illustrate immune recovery from asymptomatic SARS-CoV-2 infections. a The tissue residence signature (Tis.res.sig.) and ribosome signature (Rbs.sig.) mark CD8-GZMB and CD8-RPS27 subtypes, respectively. **b** Heat map derived from log-scaled ligand-receptor interactions counts, depicts cell-cell interactions between squamous, macrophage, neutrophil and CD8-GZMB cells in SARS-CoV-2 infection. **c-d** Dot plots of significantly altered ligand-receptor pairs using macrophages (c) or squamous epithelial cells (d) as ligand secreting cells. Selected signaling pairs highly expressed in asymptomatic infections are colored in blue, whereas those highly expressed in severely ill groups are colored in red.

Supplementary Methods

Patient recruitment and ethics approval

Ethics statement. This study was conducted according to the principles expressed in the Declaration of Helsinki. Ethical approval (2020-241) was obtained from the Research Ethics Committee of Shenzhen Third People's Hospital. All participants provided written informed consent.

Five carriers with asymptomatic SARS-CoV-2 infections were enrolled at the Shenzhen Third People's Hospital. They traveled to Shenzhen from abroad, and were immediately transferred to the isolation ward of Shenzhen Third People's Hospital when tested positive for SARS-CoV-2 during 14-day quarantined period. These cases had no clinical symptoms such as fever and cough, although CT imaging showed signs of mild pneumonia (Supplementary Table 2). The SARS-CoV-2 tests turned negative within 3~10 days in nasopharyngeal samples for these cases. Laboratory tests, including IL-6, CRP, LDH, D-Dimer, lymphocyte and neutrophil counts revealed near-normal values.

Publicly available data

ScRNA-seq data of nasopharyngeal swabs from 5 healthy controls, 8 mild and 11 severe COVID-19 patients were retrieved from European Genome-phenome Archive (https://www.ebi.ac.uk/ega/studies) with Accession number EGAS00001004481 as controls[1].

Isolation and preparation of single cells from Nose/throat swab

Nasopharyngeal cells were brushed from the nasal cavity and throat of the asymptomatic cases and placed in the RPMI1640 medium on ice and transferred to biosafety level 3 laboratory immediately. Cells in the nasal/pharyngeal swabs were dissociated by carefully pipetting. Then, cells were collected and washed by PBS by centrifuge at 1, 500 rpm centrifugation for 5 minutes. Collected cells were suspended in 200 µl DPBS and counted.

ScRNA-seq library construction

ScRNA-seq libraries were prepared according to previous protocols. In brief, the cell suspension was loaded onto a Chromium single cell controller (10X Genomics) to generate

single-cell gel beads in the emulsion (GEMs) according to the manufacturer's protocol. Reverse transcription takes place inside each GEM, after which cDNAs are pooled together for amplification and library construction. The resulting library products consist of Illumina adapters and sample indices, allowing pooling and sequencing of multiple libraries on the next-generation short read sequencer.

Cytokines measurement by Meso Scale Discovery (MSD) electrochemiluminescence assay

Plasma from 11 asymptomatic carriers was used for cytokine measurement (Extended Data Table2). Twelve healthy subjects were enrolled as the control group. Five cytokines including IL-6, IL-10, IL-12p70, IL-17A and TNF-α were detected according to the instruction (MSD, K15067L-1). In brief, 25 μl samples or standards was incubated in antibody coupled plate at room temperature for 1h, detection antibodies were added for 1h after washing by PBST. Finally, MSD GOLDTM Read Buffer B was added to read the results.

Single-cell RNA-seq data processing

The raw 5′ scRNA-seq reads were mapped against the human reference genome (GRCh38) using cellranger v3.1.0 (10X genomics). The generated feature-barcode count matrices were loaded into R (v4.0.2) with Seurat package (v3.2.2)[2] to perform batch effect correction, cell clustering and dimension reduction. We discarded cells expressing ≤ 100 genes, ≥ 6, 000 genes and ≥ 25% mitochondrial transcripts. Genes that were expressed in ≤ 3 cells were deleted. The gene expression matrices were normalized based on the total read count and log-transformed using 'NormalizeData' function. To correct batch effects, the samples were aligned using 'IntegrateData' function using the canonical correlation analysis (CCA) after choosing the top 2, 000 highly variable genes in each sample. Principal component analysis (PCA) was conducted using 'RunPCA' function followed by constructing a Shared Nearest Neighbor (SNN) Graph using 'FindNeighbors' function and cell clustering using 'FindClusters' function with a resolution of 1.2. The cell clusters were finally visualized by Uniform Manifold Approximation and Projection (UMAP) using the top 20 principal components.

Cell type annotation through canonical markers

We calculated markers expressed in each cell cluster using 'FindAllMarkers' function. The cell clusters were annotated by canonical markers (*CD68* for macrophages, *FCGR3B* for neutrophils, *CD8A* for $CD8^+$ T cells, *CD79A* for B cells, *FOXJ1* for ciliated cells, *SPRR3* for squamous cells and *SCGB1A1* for basal cells). CD8-GZMB express effector genes *GZMB*, *GNLY*, and *PRF1*. And the CD8-RPS27 subset highly expressed ribosomal genes but lacked typical effector molecules. The higher level of translation of mRNAs encoding ribosomal proteins likely helps to produce more proteins in proliferating cells.

Identification of SARS-CoV-2 reads in asymptomatic patients

The SARS-CoV-2 genome (Refseq-ID: NC045512) was added as an additional chromosome to the human reference genome (GRCh38). Besides, we added an entry summarizing the entire SARS-CoV-2 genome as a 'gene' to the GRCh38 annotation gtf file. The genome was indexed using 'cellranger mkref'. We then used cell ranger (v3.1.0; 10X genomics) to map the sequenced reads against the reconstructed reference. As a result, on the count matrices, there were no viral counts in all the asymptomatic patients. This implied that we did not detect any the SARS-CoV-2 transcripts using current sequencing method, perhaps due to the low viral load in these asymptomatic patients.

To further verify this result, we performed a Viral-Track[3] analysis to identify SARS-CoV-2 reads by combining the human (GRCh38) reference genome with thousands of virus reference genome from viruSITE.

Single cell RNA-seq signature score

Functional signatures of $CD8^+$ T cells were calculated using "AddModuleScore" function implemented in the Seurat package. Ribosome signature was calculated using genes, including *RPSA, RPS2, RPS3, RPS3A, RPS4X, RPS4Y1, RPS4Y2, RPS5, RPS6, RPS7, RPS8, RPS9, RPS10, RPS11, RPS12, RPS13, RPS14, RPS15, RPS15A, RPS16, RPS17, RPS18, RPS19, RPS20, RPS21, RPS23, RPS24, RPS25, RPS26, RPS27, RPS27A, RPS27L, RPS28, RPS29, FAU, RPLP0, RPLP1, RPLP2, RPL3, RPL3L, RPL4, RPL5, RPL6, RPL7, RPL7A, RPL7L1, RPL8, RPL9, RPL10, RPL10A, RPL10L, RPL11, RPL12, RPL13,*

RPL13A, RPL14, RPL15, RPL17, RPL18, RPL18A, RPL19, RPL21, RPL22, RPL22L1, RPL23, RPL23A, RPL24, RPL26, RPL26L1, RPL27, RPL27A, RPL28, RPL29, RPL30, RPL31, RPL32, RPL34, RPL35, RPL35A, RPL36, RPL36A, RPL36AL, RPL37, RPL37A, RPL38, RPL39, RPL39L, UBA52 and *RPL41*. Tissue residence signature was calculated using *CA10, ITGA1, ITGAE, IL2, IL10, CXCL13, CXCR6, KCNK5, RGS1, CRTAM, DUSP6, PDCD1* and *IL23R*.

Single cell TCR-seq data processing

The amino acid and nucleotide sequence of TCR chains were assembled and annotated by cellranger vdj function in CellRanger (v3.1.0). Only cells with paired TCRα and TCRβ chains were included in clonotype analysis. Cells sharing the same TCRα- and TCRβ-CDR3 amino acid sequences were assigned to the same TCR clonotype.

Analysis of DEGs

FindMarkers function in Seurat with MAST algorithm (v1.15.0)[4] was used to analyze DEGs. For each pairwise comparison, we run FindMarkers function with parameters of test.use='MAST'. Genes were defined as significantly upregulated if average natural logarithm fold change (logFC) > 0.25 and adjusted $P < 0.01$. The genes with logFC < −0.25 and adjusted $P < 0.01$ were considered significantly downregulated. The enrichment analysis of significantly upregulated and downregulated genes was conducted by clusterProfiler (v3.17.3)[5] in R. Only GO term of Biological Process was displayed.

Identification of asymptomatic patients' specific gene signatures

In each cell type, we compared the gene expression levels in asymptomatic patients to that in healthy control, mildly and severely ill patients, respectively. We take the intersection of the 3 comparisons as asymptomatic patients' specific genes. These specific genes were then enriched using clusterProfiler. The functional signature scores were calculated using genes enriched in the terms of biological processes. To reduce the variation, we normalized the gene signature score by the following formulas. $score_{new} = (score_{old} - \min(score_{old})) / (score_{old} - \max(score_{old}))$.

Cell-Cell interaction analysis

We conducted cell-cell interaction analysis utilizing cellphonedb function curated by CellPhoneDB database (v2.0.0)[6]. The significant cell-cell interactions were selected with $P < 0.01$.

Statistics

The Student's t-test (t.test in R, two-sided, unadjusted for multiple comparisons) was used for pairwise comparisons of the cell proportions and gene signature scores between different groups. Statistical difference of cytokine levels in plasma between patient groups were calculated using two-sided Student's t-test. The figures were plotted by ggplot2 (v3.3.2) package in R (v 4.0.2) and aligned using Adobe illustrator 2020.

Data availability

The raw sequence data reported in this paper have been deposited in the Genome Sequence Archive in National Genomics Data Center, Beijing Institute of Genomics (China National Center for Bioinformation), Chinese Academy of Sciences, under accession number HRA000492 that are publicly accessible at https://bigd.big.ac.cn/gsa.

Acknowledgments

We acknowledge the work and contribution of all the health providers from Shenzhen Third People's Hospital. We also thank patients for their active participation. This study was supported by the National Science Fund for Distinguished Young Scholars (82025022), Science and Technology Innovation Committee of Shenzhen Municipality (2020A1111350032), Bill & Melinda Gates Foundation, Central Charity Fund of Chinese Academy of Medical Science (2020-PT310-009), and the Natural Science Foundation of Guangdong Province of China (2020B1111340027, 2020B1111340039) and Shenzhen Bay Funding (2020B1111340075). The funders had no role in study design, data collection, data analysis, data interpretation, or writing of the report.

Conflict of interest

The authors declare no conflict of interest.

Reference

1. Chua, R.L., *et al.* COVID-19 severity correlates with airway epithelium-immune cell interactions identified by single-cell analysis. *Nat Biotechnol* **38**, 970-979 (2020).
2. Stuart, T., *et al.* Comprehensive Integration of Single-Cell Data. *Cell* **177**, 1888-1902 e1821 (2019).
3. Bost, P., *et al.* Host-Viral Infection Maps Reveal Signatures of Severe COVID-19 Patients. *Cell* **181**, 1475-1488 e1412 (2020).
4. McDavid, A., Finak, G. & Yajima, M. MAST: Model-based Analysis of Single Cell Transcriptomics. *R package version 1.16.0* **https://github.com/RGLab/MAST/**(2020).
5. Yu, G., Wang, L.G., Han, Y. & He, Q.Y. clusterProfiler: an R package for comparing biological themes among gene clusters. *OMICS* **16**, 284-287 (2012).
6. Efremova, M., Vento-Tormo, M., Teichmann, S.A. & Vento-Tormo, R. CellPhoneDB: inferring cell-cell communication from combined expression of multi-subunit ligand-receptor complexes. *Nat Protoc* **15**, 1484-1506 (2020).
